# Calcineurin/NFATc1 pathway represses cellular cytotoxicity by modulating histone H3 expression

**DOI:** 10.1038/s41598-024-65769-9

**Published:** 2024-06-26

**Authors:** Yuki Sato, Makoto Habara, Shunsuke Hanaki, Jafar Sharif, Haruki Tomiyasu, Yosei Miki, Midori Shimada

**Affiliations:** 1 Department of Veterinary Biochemistry, Joint Faculty of Veterinary Medicine, Yamaguchi University, 1677-1 Yoshida, Yamaguchi, 753-8511 Japan; 2 Developmental Genetics Group, Center for Integrative Medical Sciences (IMS), RIKEN, 1-7-22 Suehiro, Tsurumi-ku, Yokohama, Kanagawa 230-0045 Japan; 3 Department of Molecular Biology, Graduate School of Medicine, Nagoya University, 65 Tsurumai-cho, Showa-ku, Nagoya, 466-8550 Japan

**Keywords:** Histone, Transcription, Calcineurin, NFATc, Calcium, Cancer, Transcription, Phosphorylation, Cell growth, Calcium signalling

## Abstract

Excess amounts of histones in the cell induce mitotic chromosome loss and genomic instability, and are therefore detrimental to cell survival. In yeast, excess histones are degraded by the proteasome mediated via the DNA damage response factor Rad53. Histone expression, therefore, is tightly regulated at the protein level. Our understanding of the transcriptional regulation of histone genes is far from complete. In this study, we found that calcineurin inhibitor treatment increased histone protein levels, and that the transcription factor NFATc1 (nuclear factor of activated T cells 1) repressed histone transcription and acts downstream of the calcineurin. We further revealed that NFATc1 binds to the promoter regions of many histone genes and that histone transcription is downregulated in a manner dependent on intracellular calcium levels. Indeed, overexpression of histone H3 markedly inhibited cell proliferation. Taken together, these findings suggest that NFATc1 prevents the detrimental effects of histone H3 accumulation by inhibiting expression of histone at the transcriptional level.

## Introduction

In eukaryotes, the nucleosome consists of a histone octamer, 146 bp DNA wrapped around the octamer, and linker DNA. The histone octamer consists of two copies each of the histones H2A, H2B, H3, and H4. This structure is known as the nucleosome core particle and is a fundamental subunit of the chromatin. Histones include canonical histones (H3, H4, H2A, and H2B) and their variants (e.g., H3.3, H2A.Z). Canonical histones are upregulated in S-phase and package newly synthesized DNA^1^. Stem-loop binding protein (SLBP), a regulator of canonical histone mRNA, is known to be involved in replication-dependent processing of histone mRNAs, including nuclear export and translation^2–4^. On the other hand, since excess histones are toxic to the cell, histone synthesis is immediately halted with the termination of DNA replication^5^. In yeast, excess histones in the cell are known to induce mitotic chromosome loss^6^, increased DNA damage response and genomic instability^7^. Rad53 prevents the damaging effects of excess histones by degrading them^8^. Thus, histone expression levels are tightly regulated in cells to prevent the cytotoxic effects of excess histone. Ams2^7^, Spt10, and Spt21^9^ in yeast and OCA-S, Oct-1, and HiNF-P in humans are known to promote transcription of core histone genes in S phase^10–14^. On the other hand, Wee1 is known to repress histone transcription at the end of the S phase^15^. Ams2 and Wee1 in fission yeast, and Spt10 and Spt21 in budding yeast, have been shown to regulate histone transcription.

In humans, the nuclear factor of activated T cells (NFAT) family comprises five distinct genes: NFAT1 (also known as NFATc2), NFAT2 (also known as NFATc1), NFAT3 (also known as NFATc4), NFAT4 (also known as NFATc3), and NFAT5 (also known as TonEBP). NFAT exists in its phosphorylated inactive form in the cytoplasm. Upon influx of calcium ions into the cytoplasm, calcineurin and calmodulin complexes dephosphorylate NFAT, allowing its dephosphorylated form to enter the nucleus and regulate the expression of various genes^16,17^. Within the NFAT family, NFATc1 has several functions in the regulation of cancer cells. NFATc1 is known to positively act in the formation of a variety of cancers, including prostate^18^, colorectal^19^, and pancreatic^20^ cancers, it has also been reported to act as a tumor suppressor^21^. Indeed, calcineurin and NFATc1 depletion inhibits cell cycle progression in several types of cancer cells^20,22^. Dephosphorylation of NFATc1 by calcineurin facilitates not only its nuclear translocation but also increases its stability^23^. Furthermore, we previously reported that calcineurin promotes the stability of cyclin D1^22^, ERα^24^, EGFR^25^, c-Myc^26^, and FOXO1^27^ by dephosphorylation.

In this study, we found that treatment with a calcineurin inhibitor increases histone protein levels and identified NFATc1, which acts downstream of the calcium pathway, as a transcription factor that represses histone H3 transcription. Indeed, overexpression of histone H3.1 markedly inhibited cell proliferation, revealing a novel repressive mechanism by which NFATc1 escapes the detrimental effects of excessive amounts of histones.

## Results

### Calcineurin down-regulates the expression of H3.1 and H3.3

**Figure 1:**
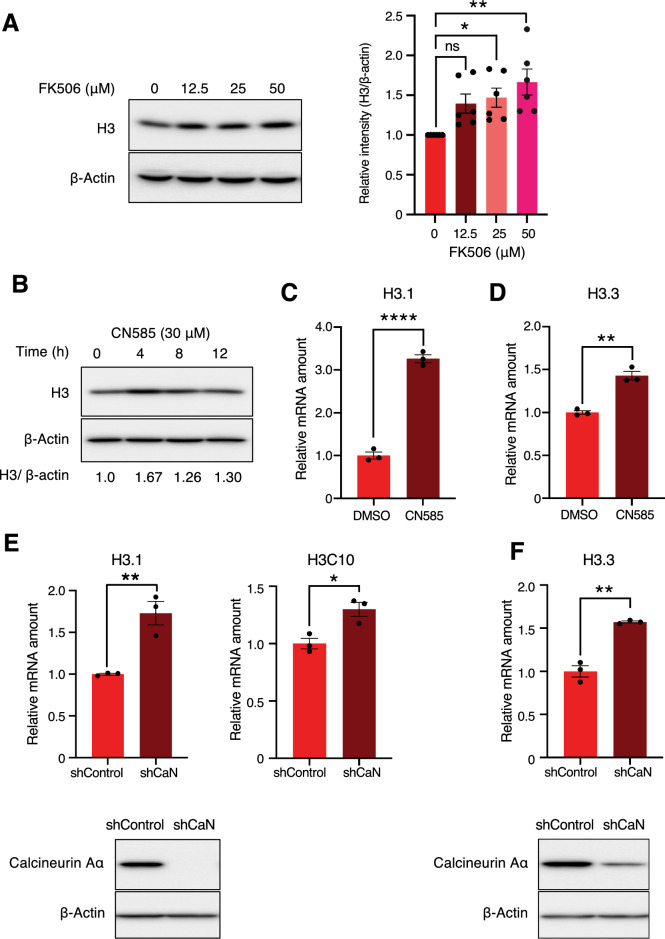
Calcineurin downregulates the expression of histone H3 (**A**) MCF7 cells were treated with the indicated concentrations of FK506 for 6 h, after which total cell lysates were prepared and immunoblotted with the indicated antibodies. The graph shows the H3 intensity relative to β-actin from immunoblot analysis. Data are expressed as the mean ± SEM of six independent experiments. ns: not significant, **P < 0.01, *P < 0.05 (two-sided, unpaired *t*-test); (**B**) MCF7 cells were treated with 30 μM CN585 for the indicated time periods, after which total cell lysates were prepared and immunoblotted with the indicated antibodies. (**C**,**D**) RT-qPCR analysis of H3.1 (**C**) and H3.3 (**D**) mRNA levels in MCF7 cells treated with CN585 or DMSO. For qPCR analysis, 18S rRNA was used as a control for the normalization of mRNA data. Data are expressed as mean ± SEM of three independent experiments. ****P < 0.0001 **P < 0.01(two-sided, unpaired *t*-test) (**E**,**F**) RT-qPCR analysis of H3.1, H3C10 (E) and H3.3 (F) mRNA in MCF7 cells expressing calcineurin A–α or luciferase shRNAs. For qPCR analysis, 18S rRNA was used as a control for the normalization of mRNA data. Data are analyzed as in C and D. **P < 0.01 *P < 0.05 (two-sided, unpaired *t*-test) The figure below shows the efficiency of calcineurin knockdown in MCF7 cells, collected and total cell lysates were immunoblotted with the indicated antibodies. Full uncropped blots are available in Supplemental Figure S4.

FK506 (tacrolimus), a widely used immunosuppressant, forms a complex with FK506 binding protein12 (FKBP12), which complex then binds to and inhibits the activity of calcineurin, a calcium/calmodulin-dependent protein phosphatase^28^. We performed proteomics analysis of FK506-treated cells and we found that more than 200 proteins were upregulated by FK506 treatment (Table S1). Since four of them were histones and it remains unclear how histone expression is regulated in mammalian cells, we focused on histone in this study. Mass spectrometry revealed that H3.2 was upregulated following FK506 treatment. However, it should be noted that the amino acid sequences of H3.1, H3.2, and H3.3 are highly similar (one amino acid variation between H3.1 and H3.2, and 3 amino acid variations between H3.2 and H3.3). Therefore, we cannot fully rule out the possibility that while the H3 protein upregulated in our mass-spec experiment was identified as H3.2, a part of the tags from H3.1 and H3.3 could have been incorrectly assigned to H3.2. As shown in Fig. 1A, H3 was increased by FK506 treatment in a concentration-dependent manner when an H3 antibody, which primarily recognizes H3.1, was used. Therefore, we further investigated the regulation of H3.1 expression. Given that FK506 also inhibits FKBP12, we used CN585, a specific inhibitor of calcineurin phosphatase activity^29^. We found that addition of CN585 also increased histone H3 protein levels (Fig. 1B). In addition, real-time PCR analysis showed that the expression levels of H3.1 (H3C3, H3C6, and H3C11) and H3.3 (H3F3A) mRNAs were upregulated in CN585-treated MCF7 cells (Fig. 1C and 1D). We also examined the mRNA levels of histones when calcineurin was depleted. We knocked down catalytic subunit of calcineurin using lentivirus-delivered short hairpin RNA (shRNA)^22^. The expression levels of H3.1, H3C10, and H3.3 mRNA were increased in calcineurin depleted MCF7 cells (Fig. 1E,F). Based on these findings, we hypothesized that calcineurin is involved in the downregulation of histone H3 expression.

### NFATc1 down-regulates the expression of H3.1 and H3.3

**Figure 2:**
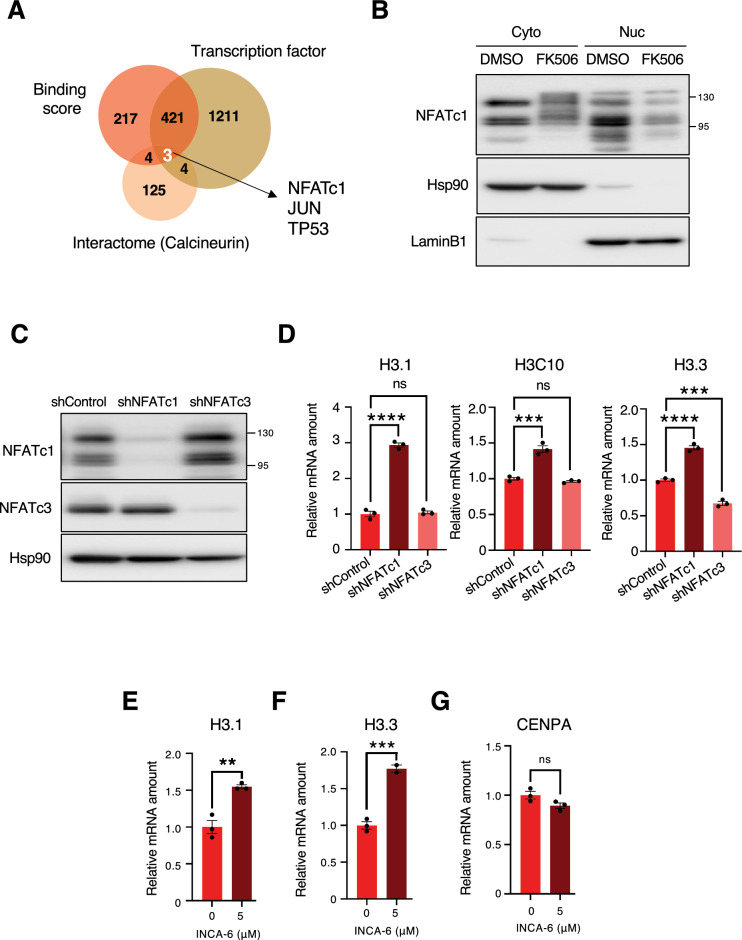
NFATc1 downregulates the expression of histone H3 (**A**) Isolation of factors that regulate histone H3 mRNA expression. From a comprehensive database analysis, we isolated three groups of indicators: Transcription factor that binds to the transcription start site (TSS) ± 500 bp of histone H3, factors known as transcription factors, factors interact with calcineurin from the BioGRID database. (**B**) MCF7 cells were treated with DMSO or 50 μM FK506 for 24 h and fractionated into cytosol and nuclear fractions. Each fraction was subjected to immunoblotting using the indicated antibodies. (**C**) Lentivirus-infected MCF7 cells were cultured in the presence of Dox to induce the expression of NFATc1 and NFATc3 shRNA. Cell lysates were prepared and analyzed by immunoblotting with the indicated antibodies. D) RT-qPCR analysis of H3.1, H3C10, and H3.3 mRNA in MCF7 cells expressing NFATc1, NFATc3, and control shRNAs. For qPCR analysis, TBP was used as a control for the normalization of mRNA data. Data are expressed as mean ± SEM of three independent experiments. ns: not significant, ****P < 0.0001, ***P < 0.001(one-way ANOVA) E,F,G) RT-qPCR analysis of H3.1, H3.3, and CENPA mRNA in MCF7 cells treated with the indicated concentrations of INCA-6 for 27 h. For qPCR analysis, 18S rRNA was used as a control for the normalization of mRNA data. Data were analyzed as described in D. ns: not significant, ***P < 0.001,**P < 0.01 (one-way ANOVA) Full uncropped blots are available in Supplemental Figure S5.

**Figure 3:**
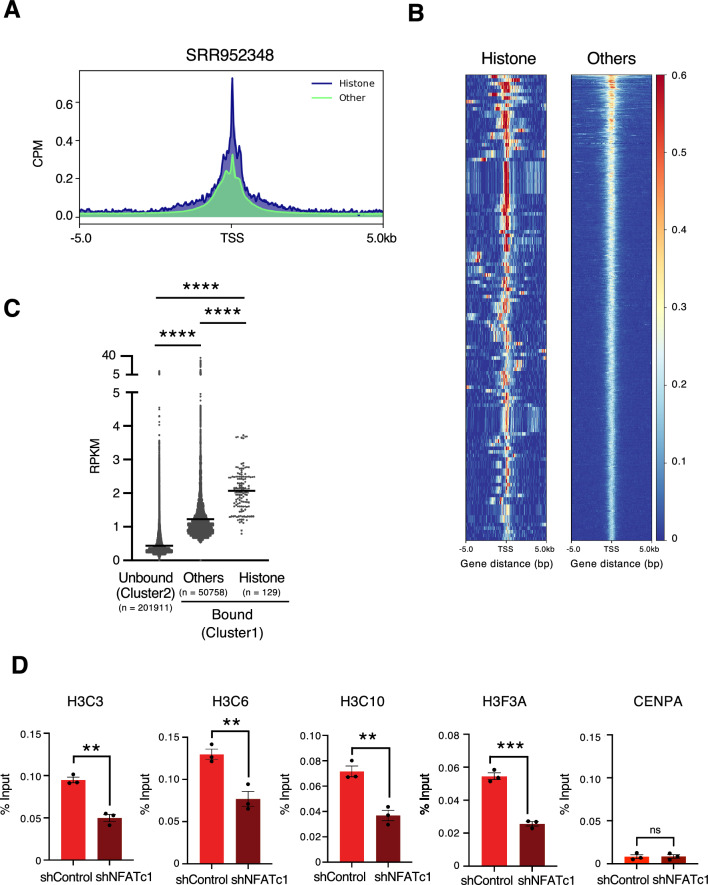
NFATc1 binds to histone promoter regions (**A**,**B**) Heatmap and profile plot showing NFATc1 occupancy 5 kb around NFATc1-bound TSS in histone-coding genes and other genes analyzed by ChIP-Seq in HUVEC. (CPM, Count per million) (**C**) RPKM of 5 kb around each TSS was analyzed using ChIP-Seq of NFATc1 in HUVEC. RPKM, reads per kilobase per million mapped reads. (**D**) ChIP-qPCR analysis of NFATc1 recruitment to the promoter regions of H3C3, H3C10E, H3C11H, H3F3A, and CENPA in MCF7 cells transfected with shNFATc1 or shControl. ***P < 0.001,**P < 0.01 (two-sided, unpaired *t*-test).

Little is known about the transcription factor of histone H3 in human. First, we searched for transcription factors that may downregulate histone expression levels downstream of calcineurin and identified NFATc1, JUN, and TP53 based on the following three criteria: (1) factors that bind to the transcription start site (TSS) ± 500 bp of histone H3 genes (H3C1,2,3,4,6,7,8,10,11,12,13,14,15) from ChIP-Atlas analysis^30,31^; (2) factors known as transcription factors^32^; and (3) factors that interact with calcineurin from the BioGRID database^33^ (Fig. 2A). Subsequently, treatment with FK506 decreased the cytoplasmic and nuclear levels of NFATc1 (Fig. 2B), which is consistent with a recent report that calcineurin inhibits NFATc1 degradation^23^. Treatment with FK506 had little effect on the amount of Jun (Fig S1A, S1B), a known substrate of calcineurin. Since p53 is not known to be a direct target of calcineurin, we examined the possibility that NFATc1 is involved in the transcriptional control of histones downstream of calcineurin. Real-time PCR analysis confirmed an increase in the mRNA expression levels of H3.1, H3C10, and H3.3 upon NFATc1 knockdown in MCF7 cells (Fig. 2C,D). We found that NFATc3 had no effect on H3.1 and H3C10 histone mRNA expression, but H3.3 mRNA expression was reduced in shNFATc3 cells for unknown reasons. The expression of H3.1 and H3.3 mRNA was increased by INCA-6 treatment, which inhibits the interaction between NFAT and calcineurin^34^, whereas the expression of CENPA mRNA, one of the major H3 variants, was not increased by INCA-6 treatment (Fig. 2E–G). Consistent with this, we found that NFATc1 did not bind to the promoter region of CENPA by ChIP-sequence analysis (Fig S2C) and ChIP-qPCR (Fig. 3D). These results indicate that NFATc1 is a transcription factor that negatively regulates histone H3 gene transcription but not CENPA transcription.

### NFATc1 binds to histone genes

To determine whether NFATc1 binds to the promoter region of histones, we reanalyzed publicly available chromatin immunoprecipitation followed by sequencing (ChIP-seq) results (GEO accession GSM1208345) for NFATc1 in human umbilical vein endothelial cells (HUVEC)^35^. First, the transcription start sites to which NFATc1 binds were identified by k-means clustering of the ± 5 kb transcription start site (bound, cluster1) (Fig S2A). Comparison of the NFATc1 binding profiles of histone-containing genes, which encompass not only H3, but also other histone genes (histones) and other genes (others) for the identified transcription start sites, showed higher NFATc1 occupancy in the histone gene group (Fig. 3A,B). Furthermore, for the "unbound", "bound-others", and "bound-histone" transcription start site + /- 5 kb, comparison of the number of mapped reads (RPKM) showed predominantly higher values in the histone gene group (Fig. 3C). Indeed, visualization of the peaks on Integrative Genomics Viewer (IGV)^36^ also confirmed that NFATc1 peaks were observed on the histone gene (Fig S2B). ChIP-quantitative PCR (ChIP-qPCR) with an NFATc1 antibody showed that NFATc1 knockdown reduced NFATc1 binding to histone promoters but had no effect on CENPA promoter (Fig. 3D). These results indicate that NFATc1 binds to the promoter region of histone H3.

### Increased calcium signaling decreases histone mRNA expression

**Figure 4:**
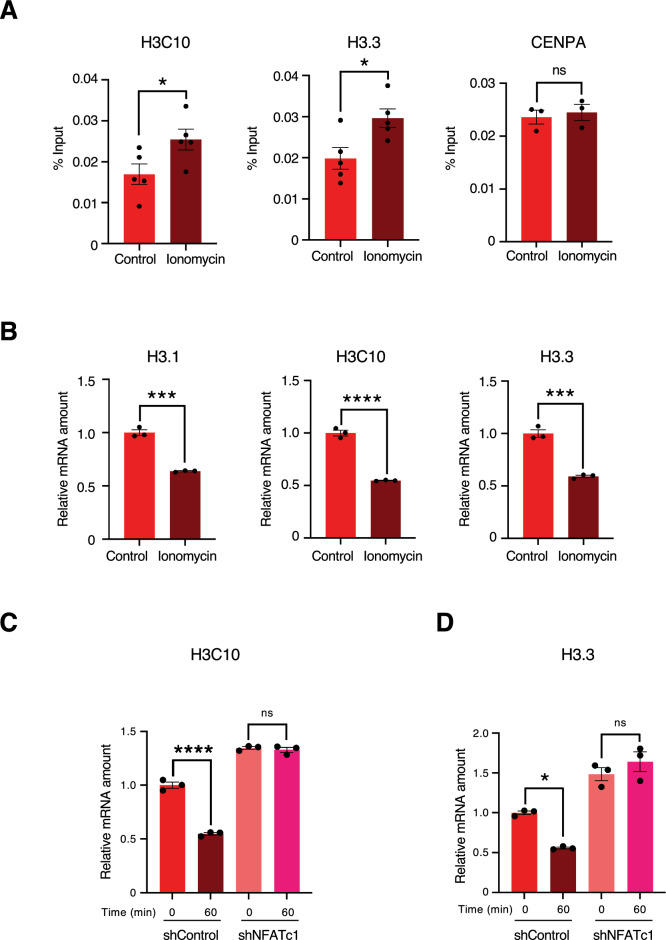
Histone transcription is suppressed by Ca^2+^ upstream of NFATc1 (**A**) ChIP-qPCR analysis of NFATc1 recruitment to promoter regions of H3C10, H3F3A, and CENPA in MCF7 cells treated with 1 µM ionomycin for 1 h. Data are expressed as mean ± SEM of five independent experiments. ns: not significant, *P < 0.05 (two-sided, unpaired *t*-test). (**B**) RT-qPCR analysis of H3.1, H3C10, and H3.3 mRNA in MCF7 cells treated with ionomycin. For qPCR analysis, 18S rRNA was used as a control for the normalization of mRNA data. Data are expressed as mean ± SEM of three independent experiments. ****P < 0.0001 ***P < 0.001(two-sided, unpaired *t*-test) (**C**,**D**) RT-qPCR analysis of H3.1 and H3.3 mRNA in shNFATc1 and shControl-expressing MCF7 cells treated with 1 μM of ionomycin with the indicated time (min). For qPCR analysis, 18S rRNA was used as a control for the normalization of mRNA data. Data were analyzed as described in B. ns: not significant, ****P < 0.0001,*P < 0.05 (one-way ANOVA).

We investigated the effects of the calcium signaling pathway upstream of NFATc1 on histone mRNA expression. To demonstrate the direct repression of histone transcription by NFATc1, we performed ChIP assays with NFATc1 antibody on MCF7 cells treated with ionomycin, a calcium ionophore, to increase intracellular calcium ion concentration^37–39^. We verified that the addition of ionomycin increased NFATc1 binding to the promoter regions of histones H3C10 and H3F3A. This increase was not observed in the promoter region of CENPA, confirming the specificity of NFATc1 in the regulation of histone H3 (Fig. 4A). Next, we treated MCF7 cells with ionomycin and found that the expression of histone mRNA decreased (Fig. 4B). On the contrary, treatment with verapamil, a calcium channel inhibitor that reduces intracellular calcium ion concentration, increased histone mRNA expression (Fig S3). We further examined the levels of histone H3.1 and H3.3 in MCF7 cells treated with ionomycin in NFATc1 knockdown cells and found that the effect of the downregulation of histone by ionomycin was counteracted by the knockdown of NFATc1 (Fig. 4C,D). These results indicate that histone mRNA expression levels are downregulated by intracellular calcium ion concentrations.

### Overexpression of histone H3.1 inhibits cell proliferation

**Figure 5:**
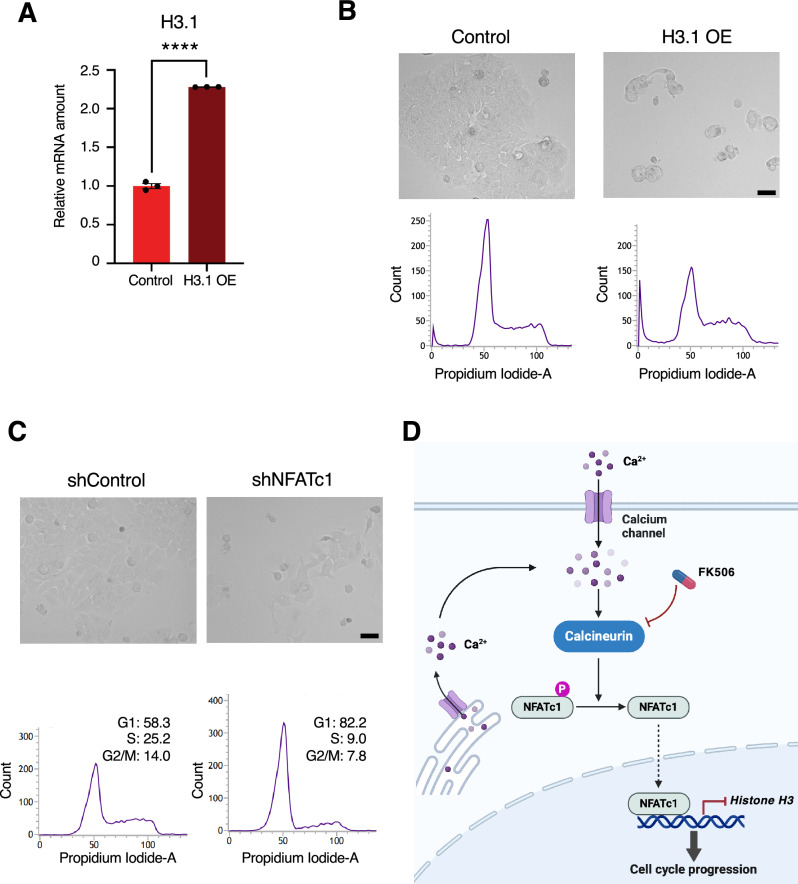
The cytotoxic effect upon the excessive amount of histone H3 (**A**) RT-qPCR analysis of H3.1 mRNA levels in MCF7 cells expressing control or H3.1. For qPCR analysis, TBP was used as a control for the normalization of mRNA data. (**B**) Typical images of cell photo in MCF7 cells expressing control or H3.1 mRNA. Bar means 40 µm. The figure below shows the cell cycle profiles obtained by FACS analysis. (**C**) Typical images of cell photo in MCF7 cells expressing control and NFATc1 shRNA. Bar means 40 µm. The figure below shows the cell cycle profiles obtained by FACS analysis. (**D**) Model of the regulation of histone H3 mRNA expression. The transcription factor NFATc1 mitigates the cytotoxic effects of excess histones by downregulating histone H3 mRNA expression. This downregulation occurs in some, but not all, histone genes. P, phosphorylation.

Based on our previous results, we showed that NFATc1 is a transcription factor that represses histone H3 mRNA expression. Finally, to investigate the physiological significance of NFATc1 in repressing histone expression, we examined the effects of overexpression of histone H3.1 mRNA in cells. First, we established cells overexpressing histone H3.1 in MCF7 cells using a lentivirus-mediated plasmid (Fig. 5A). We found that overexpression of H3.1 significantly affected cell morphology and increased the subG1 phase, as determined by fluorescence-activated cell sorting (FACS), suggesting that H3.1 overexpression causes cell death (Fig. 5B). We further examined the effect of NFATc1 knockdown on MCF7 cells and found that the cells exhibited a cytotoxic morphology and high G1/S peak, suggesting abnormal proliferation due to NFATc1 knockdown. (Fig. 5C). These results suggest that histone H3.1 overexpression negatively affects cell survival, and that appropriate histone mRNA expression mediated by NFATc1 is important for cell proliferation.

## Discussion

Excessive histone expression outside S phase is considered to be toxic, as dysregulation of histone gene expression may lead to defects in cell cycle progression, transcriptional regulation, genome stability, and DNA damage response. We show that the transcription factor NFATc1, which acts downstream of calcineurin, represses histone transcription. Calcineurin activation in response to intracellular calcium levels induces nuclear translocation of NFATc1 leading to repression of histone H3 transcription (Fig. 5D). In addition to NFATc1, we identified p53 and JUN as calcineurin interacting transcription factors that bind to histone gene promoters. JUN is stabilized upon S243 dephosphorylation mediated by calcineurin, promoting cell proliferation and tumor formation^40^. We, however, did not detect significant changes of JUN in FK506 treated cells. Therefore, we excluded the possibility that JUN mediate the suppression of histone transcription. Our analysis using the ChIP-Atlas database revealed that NFATc1 binds to multiple regions within histone genes, indicating that NFATc1 might function as a transcription factor involved in the suppression of multiple histone genes, in addition to H3. It remains to be determined whether NFATc1 is involved in suppressing the expression of core histones and histone variants other than H3.

Since multiple NFAT binding motifs are found several hundred base pairs upstream of the histone TSS, NFATc1 is thought to bind to histones via these binding motifs. NFAT represses the transcription of genes such as IL-2 and TNF-α by interacting with PPARγ, Mrj, Foxp3, and ICER through its DNA-binding domain (DBD) and NFAT-homology region (NHR)^41^. Histones may be regulated by a similar mechanism. Indeed, disruption of NFAT-mediated regulation leads to solid tumors of epithelial origin, lymphoma, and lymphoid leukemia^42^. Thus, the NFAT/CaN pathway is important for the maintenance of cellular homeostasis. The present study suggests that NFAT contributes to proliferative potential via transcriptional repression of histones. Regarding the expression of NFATc1 itself, we have recently published that dephosphorylation of NFATc1 by calcineurin not only facilitates nuclear translocation of NFATc1 but also spares it from ubiquitin-mediated degradation by Skp2^23^. Thus, proper histone transcriptional regulation via the Calcineurin-NFATc1 pathway, which is activated by increased intracellular calcium levels, may be important for cellular homeostasis.

Transcription of histone genes are mediated by NPAT, the mammalian counterpart of Spt21, specifically during the S phase. NPAT is phosphorylated by Cyclin E-Cdk2 at the G1/S transition, and acts as a scaffold protein to facilitate the recruitment of other transcription factors and co-activators to histone gene promoters^43,44^. Thus, NPAT is involved in initiating the transcription of histone genes. In contrast, U7 snRNP is known to repress replication-dependent histone gene transcription during the cell cycle, in particular outside S phase, by interacting with hnRNP protein U-like 1 (hnRNP UL1)^45^. Wee1 phosphorylates H2B-Tyr37, which prevents RNA polymerase II from binding to the genomic region of the histone, but HIRA binds and represses transcription of histone genes. Therefore, the mechanism of histone gene transcriptional repression by NFATc1 is different from Wee1.

Overexpression of histone H3.1 suppresses cell proliferation, indicating that regulation of the appropriate amount of histone transcription is important for cell proliferation. There are various reports on the effects of excess histones. In yeast, positively charged histones interact electrostatically with negatively charged intracellular molecules such as nucleic acids^46^. In addition, free histones bind nonspecifically to DNA, compete with homologous recombination (HR) machinery, and interfere with HR-mediated repair^47^. Furthermore, excess histones are known to induce whole-genome duplication, contribute to genomic instability^48^, and cause chromosome loss by disrupting the correct deposition of CENP-A^49^. Thus, excess intracellular histones may exert cytotoxic effects through a variety of mechanisms. In contrast, reduced histone levels in yeast cause significant genomic instability^50^. Reduction in total histone levels may promote mutations in important oncogenic drivers, leading to tumor progression and metastasis^51^.

Roles for core histone and linker histone for chromatin compaction has been reported^52,53^. It is therefore plausible that ectopic overexpression of various histone molecules, as we have shown in this study, might lead to increased chromatin compaction. Cellular calcium levels may counteract such chromatin compaction by repressing expression of histone genes, and consequently promoting transcription. Our findings therefore support the idea that there is a link between intracellular calcium levels and histone gene transcription. The detailed mechanism should be elucidated in the future.

In this study, we show that the transcription factor NFATc1, which acts downstream of calcineurin, represses histone transcription. Previous reports have shown that in primary human gastric, brain, breast, colon, liver, and head and neck cancer tissues and tumor cell lines, H3.2 (H3C14), is overexpressed compared to its normal counterparts of different origins; in gastric cancer, EGF-stimulated EGFR increases FOXC1 levels, which enhances H3C14 transcription. Increased H3C14 expression alters the epigenetic landscape, which positively or negatively regulates DNA accessibility, resulting in different cellular phenotypes^54^. Histone H2A (H2AC20) mRNA levels are increased in breast cancer cells and increased H2AC20 expression correlates with higher proliferation^55^. Furthermore, it has been reported that mutations in histones are associated with malignant transformation of various cancers. For example, somatic mutations in histone H3, H3K27M and G34W/L, contribute to glioblastoma^56–58^, whereas H3K36M contributes to chondroblastoma and giant cell tumors of the bone^59,60^. Therefore, suppression of histone mRNA expression via activation of the calcium pathway may be a new therapeutic target in several cancers.

## Methods

### Cell culture and reagents

MCF7 (HTB-22, ATCC) and HEK293T (632,180, Takara) cells were cultured in Dulbecco's Modified Eagle Medium (DMEM) (044–29,765; Wako) supplemented with 10% Fetal Bovine Serum (FBS) (173,012; SIGMA) and antibiotics (15,240,062; Thermo Fisher Scientific). The cells were treated with FK506 (063–06,191; Wako), CN585 (207,003; Merck), INCA-6 (ab145864; abcam), ionomycin (095–05,831; Wako), and verapamil (222-00,781; Wako). Only verapamil was dissolved in water, and all others were dissolved in dimethyl sulfoxide (DMSO). FK506 and ionomycin were used at concentrations of 50 and 1 μM, respectively. CN585, INCA-6, and verapamil were administered at the indicated concentrations.

### Construction of short hairpin RNA

We used two methods to generate lentivirus-based shRNA constructs. For shJUN creation, we used the NEBuilder® HiFi DNA Assembly Cloning Kit. In this method, the oligo was diluted in TE buffer (10 mM Tris–HCL, 0.1 mM EDTA and 50 mM NaCL) and incubated with CS-RfA-ETBsd vector and NEBuilder® HiFi DNA Assembly Master Mix at 50 °C for 1 h to produce the CS-RfA-ETBsd shJUN vector. For other constructs, we used the method described below. A 21-base shRNA-coding fragment with an ACGTGTGCTGTCCGT loop was introduced into the pENTR4-H1 vector and digested with BglII and XbaI. The pENTR4-H1-shRNA vectors were incubated with CS-RfA-ETBsd or CS-RfA-ETHyg vectors and Gateway LR Clonase Enzyme Mix (Invitrogen) for 2 h at 25 °C to produce the shRNA vector. The oligo sequences are listed in Table S2.

### Transfection

Lentivirus generation and infection were performed as described previously^45^. Lentiviruses expressing the respective genes were generated by co-transfection of 293 T cells with lentiviral-packaging vectors (1.54 μg of Pax2 and 0.86 μg of pMD2) and 2.0 μg of the respective CS-RfA-shRNA, using 8.8 μg of polyethylenimine (PEI) MAX**®** (24,765–1; Polysciences Inc., pH 7.0). Two days after transfection, the virus-containing supernatants were collected and filtered. The cells were then transduced with each lentivirus in the presence of 5 μg·mL^-1^ polybrene (H9268; Sigma-Aldrich) in standard culture medium for 24 h. Infected cells were treated with 1 μg·mL^-1^ puromycin (P7255; Sigma-Aldrich), 10 μg·mL^-1^ blasticidin (022–18,713; Wako), or 200 μg·mL^-1^ hygromycin (H3274; Sigma-Aldrich) for 2 days. To express the inducible gene, Doxycycline (D9891; Sigma-Aldrich) was added to the medium at a concentration of 1 μg·mL^-1^. For transient overexpression of plasmids, the cells were transfected with the corresponding plasmids using polyethylenimine (PEI; Polysciences Inc., pH 7.0). Briefly, cells at 50–70% confluence were incubated for 24 h with PEI and DNA. DNA–lipid complexes were diluted in Opti-Minimal Essential Medium (Opti-MEM; Gibco) and incubated for 30 min before being added to the cells. After transfection, the cell medium was replaced with serum medium. Cells were analyzed 48 h after transfection.

### Cell cycle analysis

Cell preparation for FACS and analysis of cell cycle were performed as described previously^61^. The cells fixed in 70% ethanol were then washed once with PBS, treated with RNase, and stained with propidium iodide. Flow cytometry was performed on a FACSVerse flow cytometer (BD Biosciences). The cell cycle profile was analyzed using BD FACSuite software v1.0.6 (BD Biosciences).

### Immunoblotting

The collected cells were washed with ice-cold PBS, resuspended in sample buffer (2% SDS, 10% glycerol, 100 μM DTT, 0.01% BPB, and 50 mM Tris–HCl at pH 6.8), and boiled for 5 min. Raw digital images were captured using a ChemiDoc Imaging System (Bio-Rad). The bands of the target protein were quantified using Image Lab Software 6.1 (Bio-Rad) and normalized to that of β-actin. The antibodies used in this study are listed in Table S3.

### Real-Time PCR

RNA extraction was performed using the RNeasy Plus Mini Kit (QIAGEN). A total of 800 ng of RNA was reverse-transcribed using 5 × RT Master Mix (FSQ-201, TOYOBO). Quantitative real-time PCR was performed using THUNDERBIRD® NEXT SYBR® qPCR Mix (QPX-201, TOYOBO) and a CFX96 Touch Real-Time PCR Detection System (Bio-Rad). The expression levels were normalized to 18S rRNA and TBP expression. The primer sequences are listed in Table S4.

### ChIP-qPCR analysis

ChIP-qPCR analysis was performed as previously described ^24^. Cells were fixed with 2 mM ethyleneglycol-bis-succinimidyl-succinate (21,565, Thermo Fisher Scientific) for 15 min and then with 0.5% formaldehyde for 5 min at room temperature. Fixation was terminated by the addition of glycine at a final concentration of 125 mM and incubation for an additional 3 min. The cells were isolated by centrifugation and lysed for 10 min at 4 °C in 1.5 ml of buffer LB1 (5 mM HEPES–NaOH at pH 8.0, 200 mM KCl, 1 mM CaCl_2_, 1.5 mM MgCl_2_, 5% sucrose, and 0.5% Nonidet P-40) supplemented with protease inhibitors and subjected to 20 s ON and 30 s OFF for 18 cycles of ultrasonic treatment with a Bioruptor BR-II instrument (Sonic Bio) to obtain DNA fragments of 300 to 500 bp. Antibodies against NFATc1 were bound to Dynabeads (10004D, Invitrogen) by overnight incubation at 4 °C in LB1 supplemented with 10% bovine serum albumin and then added to the samples. After overnight incubation at 4 °C, the beads were isolated and washed consecutively with Wash Buffer 1 (5 mM HEPES–NaOH at pH 8.0, 200 mM KCl, 1 mM CaCl_2_, 1.5 mM MgCl_2_, 5% sucrose, and 0.5% Nonidet P-40), Wash Buffer 2 (5 mM HEPES–NaOH at pH 8.0, 500 mM KCl, 1 mM CaCl_2_, 5% sucrose, and 0.5% Nonidet P-40), and Wash Buffer3 (10 mM Tris–HCl at pH 8.0, 1 mM EDTA). DNA was eluted from the beads in elution buffer (50 mM Tris–HCl at pH 8.0, 10 mM EDTA, and 1% SDS) and cross-links were reversed by incubation overnight at 65 °C. DNA was purified using a QIAquick PCR Purification Kit (QIAGEN) and subjected to qPCR analysis using the primers listed in Table S5.

### ChIP-Seq Analysis

Publicly available ChIP-Seq data for chromatin immunoprecipitated with the NFATc1 antibody in human umbilical vein endothelial cells (HUVEC) were obtained from the Sequence Read Archive (SRA) under accession number SRR952348^35^. Raw reads were processed using fastp version 0.23.2^62^ for trimming and quality control. Reads were aligned to the human reference genome (GRCh38) using Bowtie2 version 2.5.1^63^. SAM files were processed using samtools version 1.17^64^ and subjected to deepTools version 3.5.1^65^ to generate a heatmap and profile plot. All TSS were classified as NFATc1-bound and non-bound by k-means clustering. NFATc1-bound TSSs were divided into histone-coding genes and others, and heatmaps and profile plots were generated. A BED file describing all TSS + /- 5 kb regions was created and reads mapped to each region were counted using BEDTtools version 2.31.0^66^. Counts were normalized using the RPKM method.

### Statistical analysis

All statistical analyses were performed using the data obtained from at least three biological replicates. Statistical analyses were performed using the GraphPad Prism version 9. Quantitative data are represented as mean ± SEM and were analyzed using the unpaired t-test for the comparison of two groups or by one-way ANOVA followed by Dunnett’s test for comparisons among three or more groups. Statistical significance was set at P < 0.05.

### Database analysis to identify transcriptional suppressors of histone H3

Factors that interact with calcineurin were obtained from the BioGRID database^33^. Curated transcription factors were obtained from a previously reported list^32^. Transcription factors binding to histone H3 were obtained from enrichment analysis in the ChIP-Atlas^30,31^. Only factors that bind to the transcription start site ± 500b of histone H3 genes (H3C1,2,3,4,6,7,8,10,11,12,13,14,15) and have a binding score of 1200 or higher were selected.

### Sample preparation for LC–MS/MS analysis

To prepare the sample for LC–MS/MS measurement, we followed the previously reported method^67^. The sample was initially treated with 10 mM dithiothreitol at 50 °C for 30 min. Subsequently, alkylation was performed using 30 mM iodoacetamide in a dark environment at room temperature. The resulting mixture was then diluted four-fold with 50 mM ammonium bicarbonate and subjected to digestion using 800 ng Lys-C and 400 ng trypsin overnight at 37 °C. To extract the digested samples, an equal volume of ethyl acetate was added, followed by acidification with 0.5% trifluoroacetic acid (TFA) according to the PTS protocols^68,69^. After shaking for 5 min and centrifuging at 15,000 × g for 5 min to achieve phase separation, the aqueous phase was collected. The volume of the recovered digested sample was reduced to less than or equal to half of the original volume using a centrifugal evaporator to remove ethyl acetate completely. The remaining mixture was then desalted using C18-Stage Tips^70^. The peptides trapped in the C18-Stage Tips were eluted with 40 µL of 50% acetonitrile (ACN) and 0.1% TFA, followed by drying using a centrifugal evaporator. The dried peptides were reconstituted in 20 µL of 3% ACN and 0.1% formic acid. Finally, 2 µL of the reconstituted sample was subjected to LC–MS/MS analysis.

### LC–MS/MS and data analysis

For SWATH protein quantification, a preliminary DDA (data-dependent acquisition) set was performed as below^67^. Approximately 100 ng of peptides was directly injected onto a PicoFrit emitter (100 µm × 15 cm) packed with 120 A porous C18 particles. The peptides were separated using a 240-min gradient of acetonitrile (ACN) ranging from 3 to 40% at a flow rate of 300 nl/min using an Eksigent ekspert nanoLC 400 HPLC system (Sciex). The eluted peptides were then analyzed using a TripleTOF 5600 + mass spectrometer. MS1 spectra were collected in the m/z range of 400–1200 for 250 ms. The top 25 precursor ions with charge states ranging from 2 + to 5 + and exceeding 150 counts/s were selected for fragmentation using rolling collision energy. MS2 spectra were collected for 100 ms, and a spray voltage of 2100 V was applied.

All MS/MS files were searched against the UniProtKB/Swiss-Prot human database (Proteome ID: UP000005640, downloaded October 19, 2018, 20,410 protein entries) combined with the standard MaxQuant contaminants database (http://www.coxdocs.org/doku.php?id=maxquant:start_downloads.htm) using ProteinPilot software v. 4.5 with the Paragon algorithm for protein identification^67^. The search parameters included cysteine alkylation by iodoacetamide, trypsin digestion, and TripleTOF 5600 instrument specifications. Protein identification required a ProteinPilot unused score of 1.3 with at least one peptide having 95% confidence. Contaminating proteins were excluded from the identified proteins and peptides. The global false discovery rate for both peptides and proteins in this study was less than 1%.

SWATH DIA (data-independent acquisitions) were performed using the same gradient profile as the DDA experiments described above. Precursor ion selection was conducted in the m/z range of 400–1200 with a variable window width strategy ranging from 7 to 75 Da. Collision energy for each SWATH experiment was set at 45 eV, and 80 consecutive SWATH experiments covering the m/z range of 100–1800 were performed, each lasting 36 ms. DIA raw data were analyzed using the SWATH processing functionality embedded in PeakView software (SCIEX). Retention time recalibration between runs was achieved using peptides from PIG Trypsin and Protease I precursor Lysyl endopeptidase. The following criteria were applied for DIA quantification: peptide confidence threshold of 99%, maximum mass tolerance of 30 ppm, and maximum retention time tolerance of 6 min. Multivariate data analysis was performed using Markerview software (SCIEX).

The value shown for each protein represents the number of protein fragments detected for that particular protein divided by all the detected protein fragments in the same experiment. As the possibility that the changes observed at 12 h might be, in part, caused by the indirect effects of prolonged (12 h) FK506 treatment cannot be ruled out, we focused more on the results gained at 3 h after FK506 treatment.

### Supplementary Information

Supplementary Tables.

Supplementary Information.

Supplementary Table S1.

## Data Availability

The accession numbers of proteomics datasets are PXD042833 for ProteomeXchange and JPST002185 for jPOST. Additional data related to this paper may be requested from the authors.
