# Supplementary material for: Calcineurin/NFATc1 pathway represses cellular cytotoxicity by modulating histone H3 expression
Source: Sci Rep. 2024 Jun 26;14:14732. doi: 10.1038/s41598-024-65769-9 (PMC11208570; doi:10.1038/s41598-024-65769-9)
Supplement: Supplementary file 1 — Supplementary Tables. [file 41598_2024_65769_MOESM1_ESM.docx]

## Supporting information

## Table S2

## Target sequences for lentivirus-based shRNA.

| **Gene** | **Sequence (5’-3’)** |
| --- | --- |
| Luciferase | CGTGCGTGGAATGCTTCGA |
| PPP3CA-1 | GCGTATATGATGCCTGTATGG |
| JUN-1 | TAGTACTCCTTAAGAACACAA |
| JUN-2 | CAAACCTCAGCAACTTCAA |
| NFATc1 | GCCAGTACCAGCGTTTCAA |
| NFATc3 | GCTTACCACATCATGGATTAC |

## Table S3

## Antibodies used in this study.

| **Antigen** | **Catalog No.** | **Company** |
| --- | --- | --- |
| β-actin | ab6276 | Abcam |
| Hsp90 | sc13119 | Santa Cruz Biotechnology |
| NFATc1 | sc7294 | Santa Cruz Biotechnology |
| NFATc3 | 18222-1-AP | Proteintech |
| H3 | MABI0301 | MAB Institute, Inc. |
| JUN | sc74543 | Santa Cruz Biotechnology |
| LaminB1 | ab16048 | Abcam |

## Table S4

## Primer sequences for RT-qPCR.

| **Gene** | **Forward (5’-3’)** | **Reverse (5’-3’)** |
| --- | --- | --- |
| 18S rRNA | GTAACCCGTTGAACCCCATT | CCATCCAATCGGTAGTAGCG |
| TBP | TGTATCCACAGTGAATCTTGGTTG | GGTTCGTGGCTCTCTTATCCTC |
| H3.1 | CTTCCAGAGTTCCGCG | CGCGTTTGGCATGAATA |
| H3.3 | CTGATTCGCAAACTTCCC | AAGCACCGATAGCTGCG |
| H3C10 | ATCGCTATCGGCCTGGTACA | TTTCTGATCAGCAGCTCGGT |
| CENPA | GCTTAGCCACTTGGCTCCTAA | TGAAGCTGAGATAACGCACGA |

## Table S5

## Primer sequences for ChIP-qPCR.

| **Gene** | **Forward (5’-3’)** | **Reverse (5’-3’)** |
| --- | --- | --- |
| H3C3 | TTATCGCGCGGGACTTTTGA | GCTTCGTACGAGCCATTTGC |
| H3C6 | AATCTCTACGGCCACTTCCG | TGCTTAGTACGCGCCATAGT |
| H3C10 | TCGCCAATCCGGTTACTGTT | GCCATGCCTTCAGAGCTAGATT |
| H3F3A | CCGAAAACAGATCACACGGC | GCGTCGGCCTCTAAACAATG |
| CENPA | GGCGGAGACAAGGTTGGCTAAA | GGCTTGCCAATTGAAGTCCACAC |
