# Supplementary material for: Calcineurin/NFATc1 pathway represses cellular cytotoxicity by modulating histone H3 expression
Source: Sci Rep. 2024 Jun 26;14:14732. doi: 10.1038/s41598-024-65769-9 (PMC11208570; doi:10.1038/s41598-024-65769-9)
Supplement: Supplementary file 2 — Supplementary Information. [file 41598_2024_65769_MOESM2_ESM.pdf]

Supplementary information

Calcineurin/NFATc1 pathway represses cellular cytotoxicity by modulating histone H3 expression

Yuki Sato, Makoto Habara, Shunsuke Hanaki, Jafar Sharif, Haruki Tomiyasu, Yosei Miki, and Midori Shimada

Fig S1

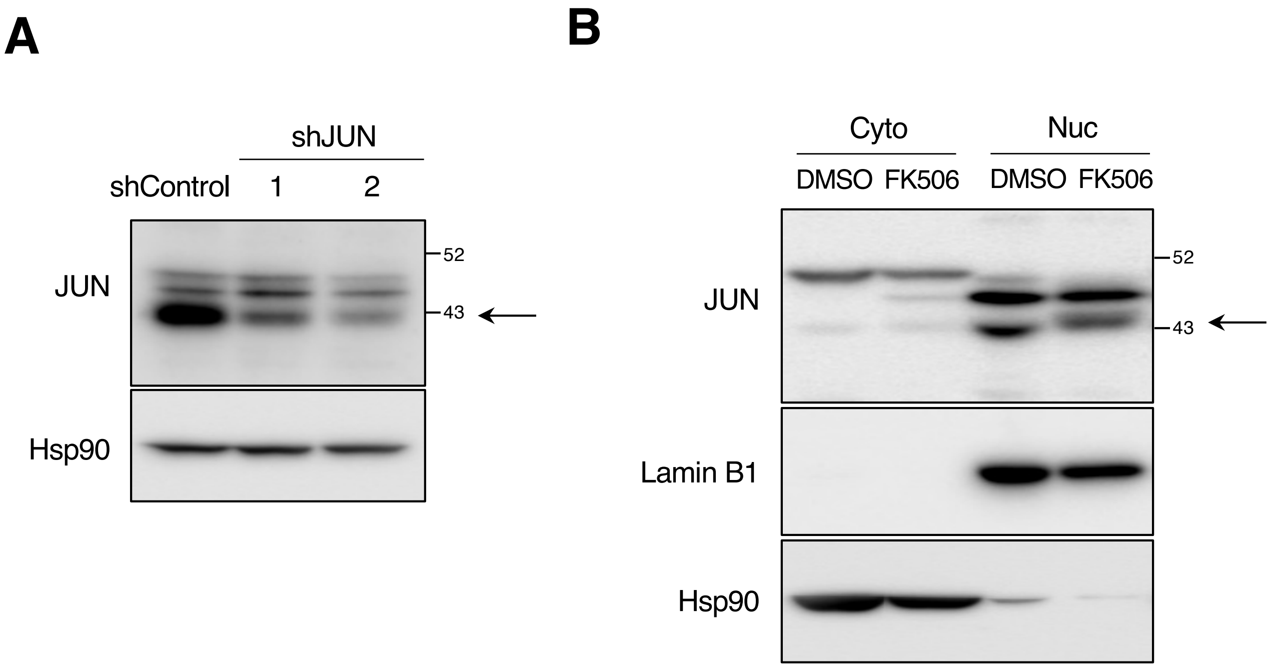

Figure S1

A) Lentivirus-infected MCF7 cells were cultured in the presence of Dox to induce JUN shRNA expression. Cell lysates were prepared and analyzed by immunoblotting with the indicated antibodies. The arrows indicate the JUN protein bands which were confirmed in the knockdown cells. **B)** MCF7 cells were treated with DMSO or 50 μM FK506 for 24 h and fractionated into nucleoplasmic, and cytoplasmic fractions. Each fraction was subjected to immunoblotting using the indicated antibodies. The arrows indicate the JUN protein bands which were confirmed in the knockdown cells.

Fig S2

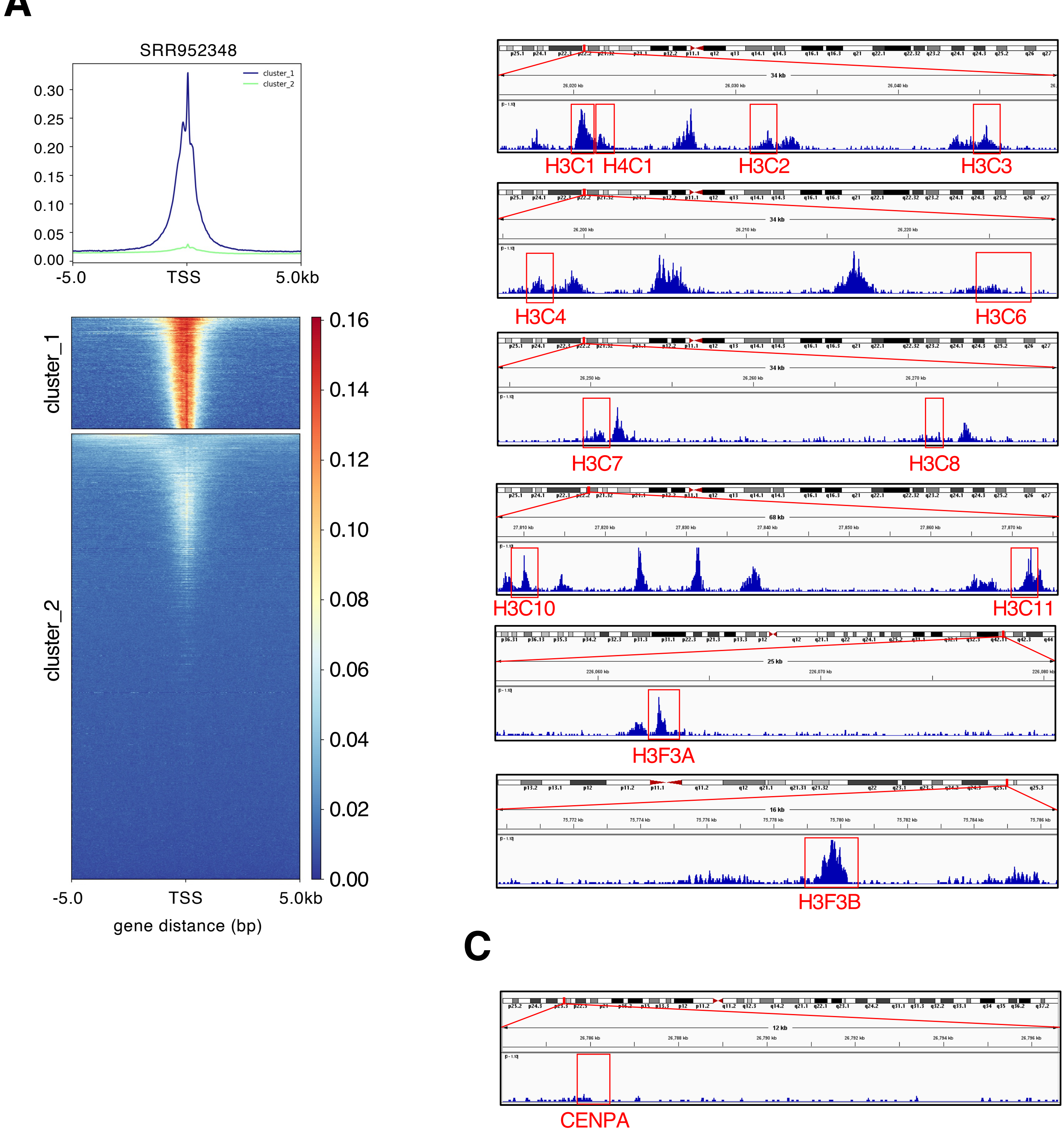

Figure S2

A) Heatmap and profile plot showing NFATC1 occupancy 5kb around the TSS analyzed by ChIP-Seq in HUVEC. All TSS were classified into two groups by k-means clustering: NFATC1-bound and non-bound, respectively. (CPM, Count per million) **B,C)** ChIP-Seq signals (peaks) of anti-NFATc1 antibody after loading BigWig files from the ChIP-Atlas were visualized on Integrative Genomics Viewer (IGV). Red boxes indicate histone H3, H4C1 (B), and CENPA (C) gene regions.

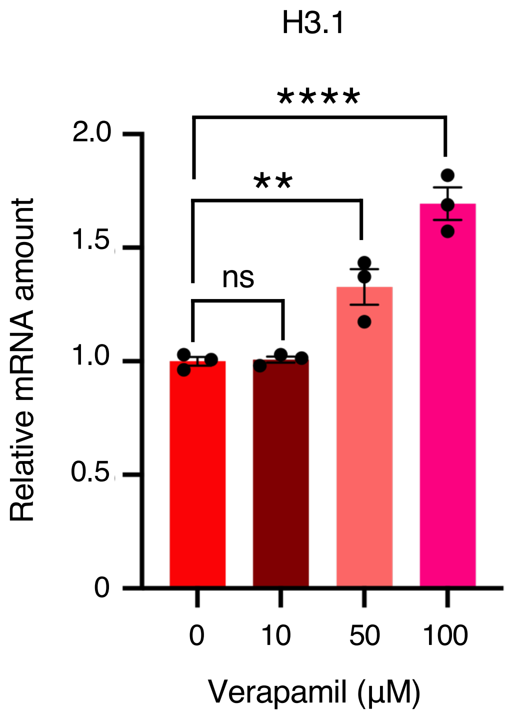

Figure S3

RT-qPCR analysis of H3.1 mRNA in MCF7 cells treated with the indicated concentrations of verapamil for 9.5 h. For qPCR analysis, TBP was used as a control for the normalization of mRNA data. Data are expressed as mean ± SEM of three independent experiments. ns: not significant, ****$P < 0.0001$, **$P < 0.0001$ (one-way ANOVA)

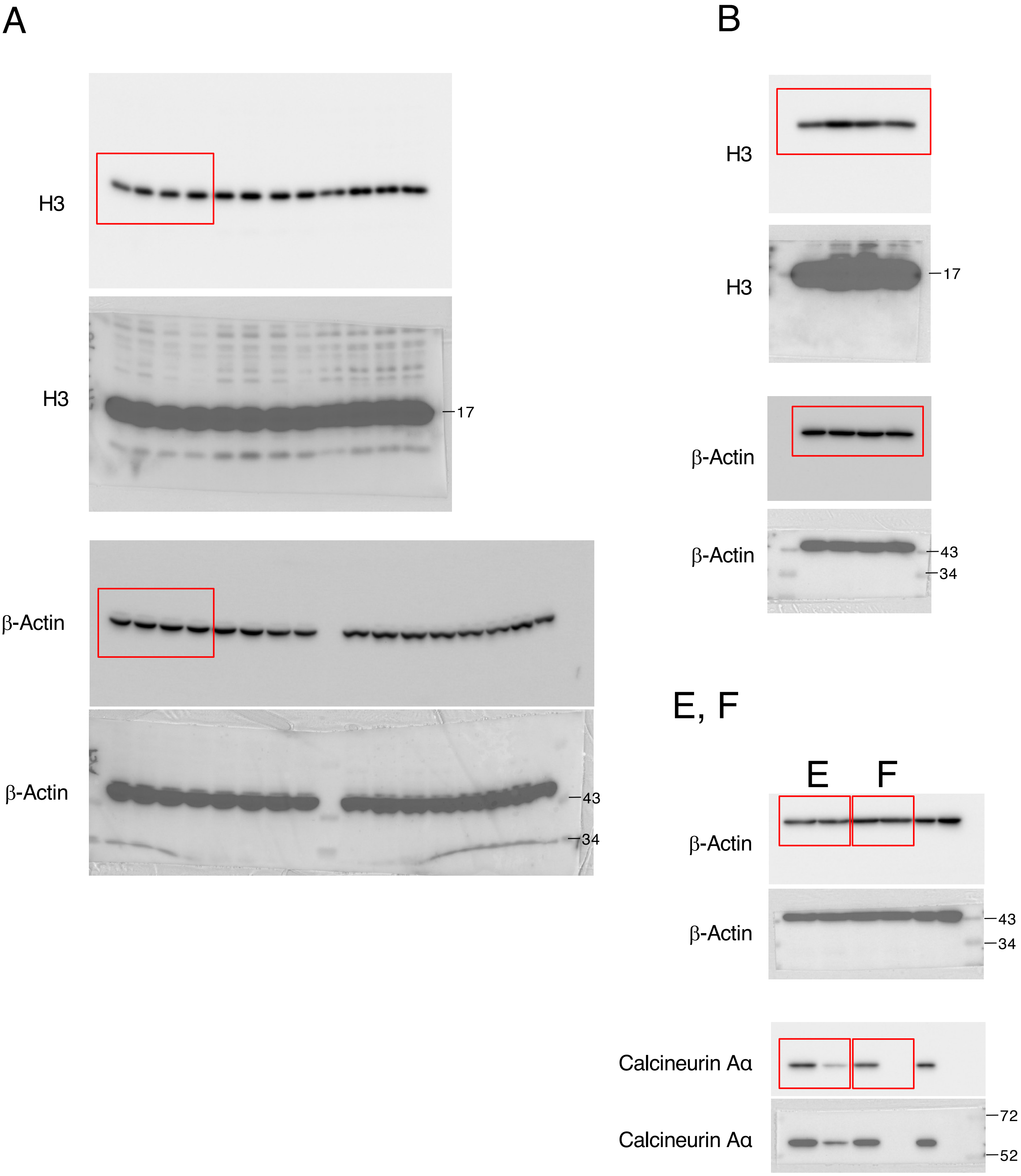

Figure S4
Full unedited images for Figure 1 are shown.

Fig S5

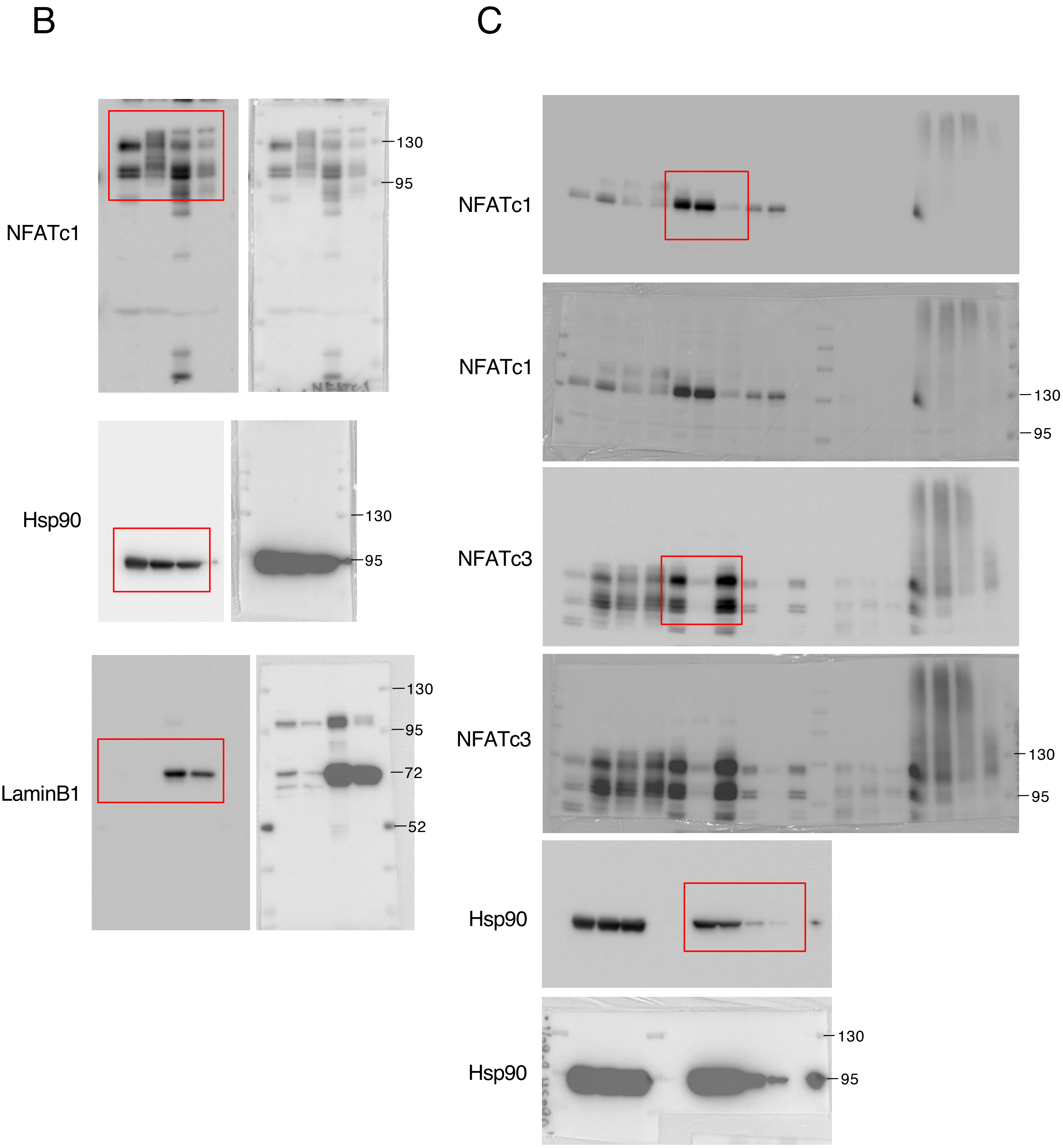

Figure S5
Full unedited images for Figure 2 are shown.

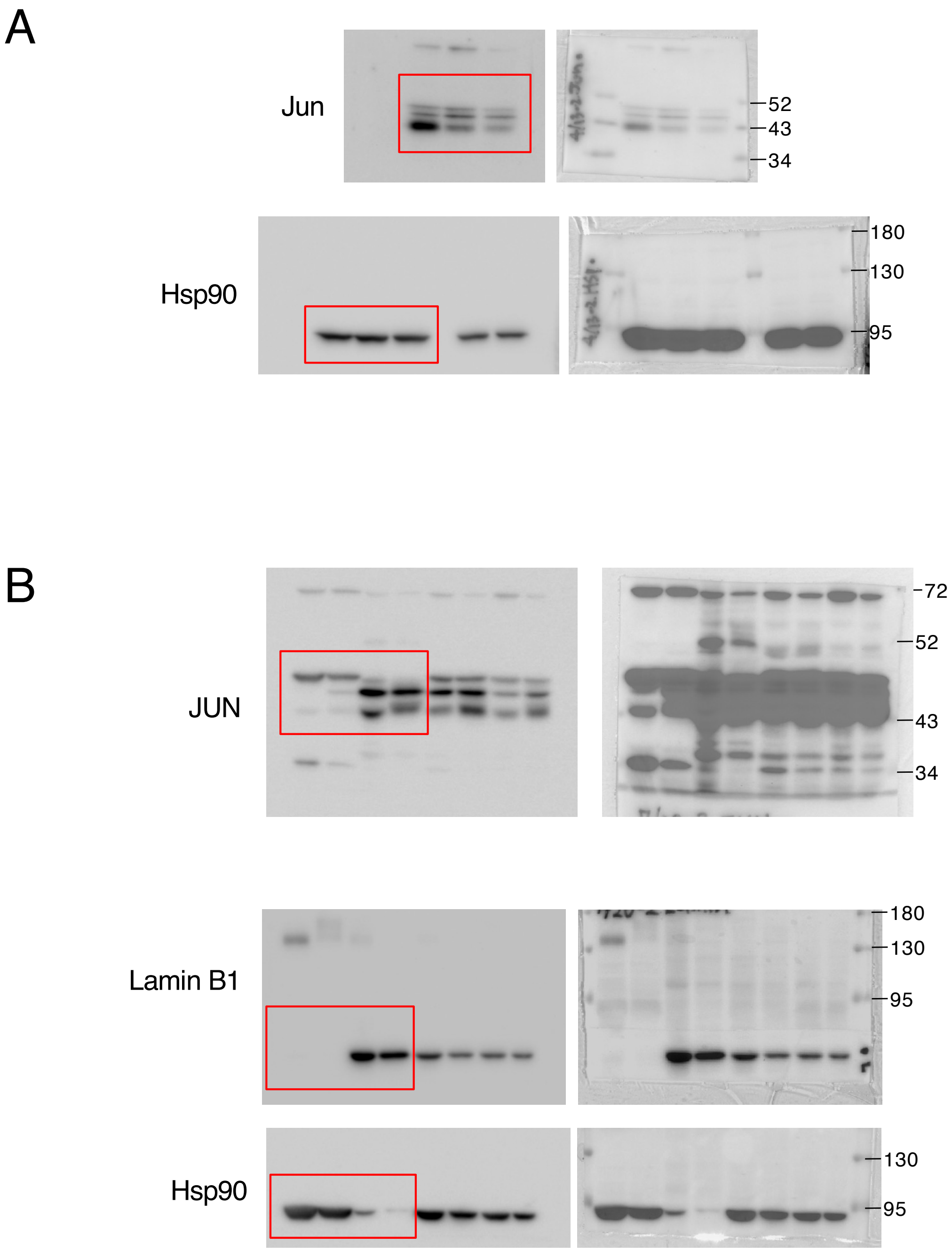

Figure S6
Full unedited images for Figure S1 are shown.
